# Local Scheduling in KubeEdge-Based Edge Computing Environment

**DOI:** 10.3390/s23031522

**Published:** 2023-01-30

**Authors:** Seong-Hyun Kim, Taehong Kim

**Affiliations:** School of Information and Communication Engineering, Chungbuk National University, Cheongju 28644, Republic of Korea

**Keywords:** edge computing, Kubernetes, KubeEdge, EdgeMesh, load balancer, microservice

## Abstract

KubeEdge is an open-source platform that orchestrates containerized Internet of Things (IoT) application services in IoT edge computing environments. Based on Kubernetes, it supports heterogeneous IoT device protocols on edge nodes and provides various functions necessary to build edge computing infrastructure, such as network management between cloud and edge nodes. However, the resulting cloud-based systems are subject to several limitations. In this study, we evaluated the performance of KubeEdge in terms of the computational resource distribution and delay between edge nodes. We found that forwarding traffic between edge nodes degrades the throughput of clusters and causes service delay in edge computing environments. Based on these results, we proposed a local scheduling scheme that handles user traffic locally at each edge node. The performance evaluation results revealed that local scheduling outperforms the existing load-balancing algorithm in the edge computing environment.

## 1. Introduction

With the development of Internet of Things (IoT) technology, various IoT sensors and devices are being deployed daily, and there is an increase in the number of artificial intelligence services that can recognize IoT user behavior patterns and situations based on the data collected from IoT devices [1,2]. In such a scenario, the user data are typically transferred to the cloud located at the center of the network and are analyzed and processed using cloud computing resources. However, cloud-based systems have a centralized structural limitation in meeting the requirements of IoT application services [3,4], which require a low response latency that is within tens of milliseconds. Edge computing was proposed to solve this problem. Edge computing reduces the response time by placing computational resources on locally distributed edge nodes instead of transmitting the data to the central cloud, thereby meeting the requirements of time-critical IoT application services [5].

A container is a unit of software that packages files such as libraries, binaries, and other configuration files required to run an application on an operating system (OS). Therefore, it provides the advantage of preventing program execution errors due to different environments such as networks, security, and build environments, thus driving them to operate stably. The container simplifies the distribution, installation, update, and deletion of IoT application services on edge nodes due to the lightness and portability of the container [6]. Moreover, various types of IoT application services can be provided simultaneously on each edge node. As such, containers are the most suitable technology for providing IoT application services in an edge computing environment. However, container orchestration is required to monitor and manage resource states via multiple edge nodes in an edge computing environment because containers can be applied only to the deployment and management of application services on a single node [7,8].

Kubernetes [9,10] is a representative container orchestration platform released by Google, which provides the deployment, resource monitoring, and management of container-based application services in multinode environments. It provides a series of processes for managing IoT application services with containers, such as creation and deletion of containers, scheduling to distribute the increased containers to appropriate nodes, and clustering such that multiple nodes can be used as a single server [11].

KubeEdge [12] is a lightweight container orchestration platform that allows container-based IoT application services to run on edge nodes. It incorporates the Kubernetes service used in cloud computing into edge computing. KubeEdge can control edge nodes in the same manner used to operate the Kubernetes cluster in the existing cloud environment and can readily distribute various IoT application services such as machine learning, image recognition, and event processing to edge nodes. EdgeMesh [13] essentially supports service discovery and proxy functions for each pod in the application. It also provides load balancing by distributing user traffic to each pod in the cluster. However, this load-balancing function has a fundamental drawback in edge computing environments. In an edge computing environment, edge nodes are geographically dispersed and the pods of the applications are also distributed throughout the edge nodes. In other words, the load-balancing function in EdgeMesh distributes the user traffic to the application pods in the cluster. However, it encounters latency when forwarding requests between edge nodes, thereby degrading the application throughput in the cluster [14]. To solve this limitation in a KubeEdge-based edge computing environment, we propose a local scheduling scheme that processes user traffic at the local node without forwarding the traffic to the remote nodes. Experimental evaluation results prove that the local scheduling scheme can provide low latency as well as improve the throughput of the cluster by suppressing the traffic forwarding in edge computing environments. 

The contributions of this study can be summarized as follows:

- To the best of our knowledge, this study is the first to evaluate the performance of KubeEdge. We conducted diverse performance evaluations regarding the amount of computational resources, in other words, the pod distribution throughout edge nodes and the delay between edge nodes.
- It was observed that the throughput of the cluster can be degraded due to traffic forwarding between edge nodes. We address the delay caused by the load balancing of EdgeMesh, which negatively impacts the performance of edge computing environments.
- To overcome the performance degradation in a KubeEdge-based edge computing environment, we propose a local scheduling scheme and compare the performance in terms of throughput and latency, which provides important lessons for operating the KubeEdge platform in an edge computing environment.

The remainder of this paper is organized as follows. Section 2 introduces related research, and Section 3 describes the basic background of KubeEdge architecture, components, and EdgeMesh. Section 4 describes the system model and the problem definition as well as the proposed local scheduling scheme. Section 5 evaluates the diverse performance of KubeEdge, such as the effect of pods and the effect of node-to-node delay between edge nodes, and compares the load-balancing scheme and EdgeMesh’s round-robin scheme in cluster performance. Finally, Section 6 concludes this paper.

## 2. Related Work

This section presents an analysis of studies related to KubeEdge and throughput improvement techniques in edge computing environments. KubeEdge was announced by Huawei [15] in 2018 as an open-source system that extends the functions of applications requiring service distribution, expansion, and management to edge hosts. Yang et al. [16] investigated artificial intelligence (AI) for networks (NET4AI) and EdgeMesh computing for networks. They extended the role of cloud to communication networks and suggested a development direction for integrated communication systems. They fused KubeEdge technology with edge computing and mesh networking [17] and proposed the KubeEdge wireless platform for dynamic application services. The platform handles various objects such as vehicles, people, and homes, connected to mesh networks, and shares computational resources. In particular, subscribers are considered mobile routers that build dynamic mesh networks while supporting computational resource sharing and mesh network subscription.

Zheng et al. [18] trained a lifelong learning model [19] to develop a lifetime thermal comfort prediction framework that predicts thermal comfort. It was developed based on KubeEdge–Sedna [20] as an edge–cloud synergy AI project at KubeEdge and was designed to automatically learn the passive functions of the existing model. Knowledge of the model, that is, meta-knowledge, can be used to predict the thermal comfort of people living indoors, which can be extended to numerous building interiors and software contexts to estimate long-term thermal comfort.

Rui Han et al. [21] proposed EdgeGossip on the KubeEdge platform, aiming to quickly obtain model accuracy and avoid low-performance deviations during iterative training in deep learning. EdgeGossip balances training time by estimating the performance of multiple edge computing platforms during iterative training. It also provides the ability to use the aggregated data points to identify areas related to the accuracy of the data entered, improving the best-effort model accuracy. EdgeGossip is implemented on the Gossip algorithm [22], and its effectiveness was demonstrated using real-time deep-learning workloads. Mutichiro et al. [23] proposed StaSA, which can satisfy the quality of service (QoS) requirements of users as an edge application. The STaSA scheduler improves cluster resource utilization and QoS in edge–cloud clusters in terms of service time by automatically assigning requests to different processing nodes and scheduling execution according to real-time constraints. The performance of the proposed scheduling model was demonstrated on the KubeEdge-based implementation. Tran et al. [24] presented the NDN network over edge computing infrastructure to provide a disaster response support system. The authors defined emergency group communication and disaster information exchange through NDN. The feasibility of the proposed system was demonstrated by implementing the KubeEdge-based infrastructure with NDN IoT devices.

With the development of container technology, studies on improving the production environment of container-based applications have been conducted. Abouaomar et al. [25] investigated resource provisioning at the network edge under latency and resource consumption constraints. By studying the frequency of resource allocation by the head of the edge node, they proposed a Lyapunov optimization framework on each edge device to reduce the number of resource allocation operations. Consequently, they validated that the proposed approach outperforms other benchmark approaches and provides low latency and optimal resource consumption. Taherizadeh et al. [26] proposed a dynamic multi-level auto-scaling technique for container-based application services, and [27,28,29] proposed Kubernetes-based resource provisioning and service quality improvement measures. Le et al. [27] address the limitation of the Kubernetes horizontal pod autoscaler, in that it is not suitable for different traffic distribution environments with real-time service demand in edge computing environments. They proposed the traffic-aware horizontal pod autoscaler to improve service quality by dynamically adjusting cluster resources according to the network traffic distribution. Nguyen et al. [28] proposed a proxy for an improved Kubernetes, referred to as RAP, which offloads latency caused by the load during load balancing to other optimal nodes. Gupta et al. [29] proposed a method to containerize and deploy deep-learning models to learn from edges and improve service quality by reducing data latency and traffic. In addition, the article EdgeX over Kubernetes [30] proposed a method to improve service quality by distributing computational resources that IoT gateways handle, given the combination of cloud computing and edge computing platforms. Choi et al. [31] proposed an intelligent service management technique that can handle large amounts of data generated by a large number of devices in real time while solving various problems such as connectivity and security in an industrialized IoT environment.

Consequently, KubeEdge has been considered a key platform for building edge computing infrastructure and providing application services. Nevertheless, comprehensive performance evaluation and analysis of KubeEdge have not been performed. In this study, we conducted an experimental performance analysis of KubeEdge in an edge computing environment. We observed that although the load-balancing feature of KubeEdge generally provides high availability and scalability of the cluster, it can degrade the performance due to delays between edge nodes. Therefore, we propose a local scheduling scheme to overcome this problem and maximize the performance of KubeEdge-based edge computing environments.

## 3. Preliminaries of KubeEdge

This section introduces the KubeEdge architecture and main components, and how it works. We also discuss EdgeMesh, which is one of the important components providing load balancing in KubeEdge.

### 3.1. KubeEdge Architecture

**Figure 1:**
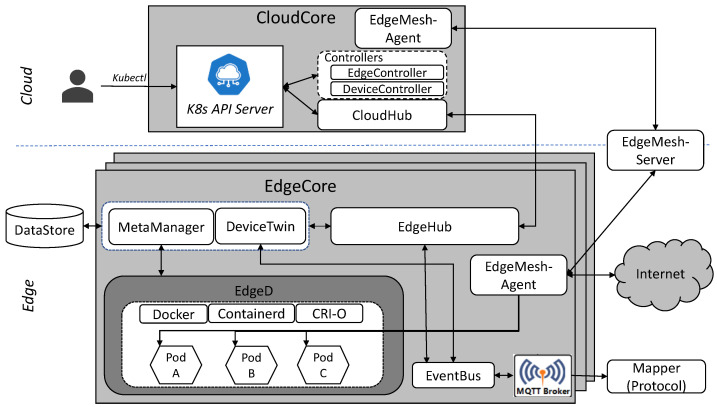
KubeEdge architecture and its components in cloud and edge.

KubeEdge [12] is a lightweight open-source edge computing platform developed under the Huawei initiative. It provides network management between edge nodes and the cloud, in addition to the maintenance of sessions when edge nodes are offline, as it aims to apply edge computing environments from the start of the design. It supports the MQTT protocol to enable resource-limited IoT edge devices to communicate efficiently. Figure 1 presents the architecture of KubeEdge, which consists of Cloud Core and Edge Core structures, unlike the Kubernetes master node and worker node structures [12]. Internet of Things (IoT) application services operate on Edge Core, which is geographically distributed in the edge layer, and Cloud Core manages application services. Edge Core consists of EdgeD, EdgeHub, EventBus, DeviceTwin, and MetaManager. EdgeD runs and manages container-based applications. It helps the administrator to deploy containerized workloads or applications at Edge Core. EdgeD provides diverse functionalities such as pod management, pod lifecycle event generator, secret management, and container runtime, as well as deployment of workloads. EdgeHub supports functions such as updating resource synchronization in the cloud and changing the state of edge devices via socket connectivity between Cloud Core and Edge Core in edge computing environments. EdgeHub acts as the communication link between the edge and the cloud. EdgeHub forwards messages received from the cloud to the corresponding module at the edge and vice versa. EventBus provides MQTT clients with functions to interact with IoT edge devices and supports Publish/Subscribe functions such as sending MQTT topics to CloudCore. DeviceTwin stores the state of IoT edge devices and synchronizes them to the cloud. It also provides query interfaces for applications. MetaManager is a message processor between EdgeD and EdgeHub. It is also responsible for storing and retrieving metadata from a database.

Cloud Core consists of controllers and CloudHub, and the controllers are composed of edge controller and device controller. Edge controller connects the Kubernetes application programming interface server (K8s API Server) and Edge Core. Edge controller adds, updates, deletes, monitors, and synchronizes events between the K8s API Server and Edge Core. Device controller is responsible for IoT device management. It synchronizes the IoT device updates from Cloud Core and Edge Core. CloudHub is a component of Cloud Core and is the mediator between controllers and the edge side. CloudHub monitors changes on Cloud Core, caches messages, and allows for communication between Edge Core and the controllers via socket communication with EdgeHub.

### 3.2. EdgeMesh

This subsection describes EdgeMesh, which is a data plane component of a KubeEdge cluster. EdgeMesh [13] provides service discovery and traffic proxy functionality within the KubeEdge cluster, in addition to the high availability of KubeEdge by connecting edge nodes using LibP2P [32]. In the case of Intra-LAN, communication between edge nodes is provided through direct access. For Cross-LAN, communication between edge nodes is supported via a tunneling technique using hole punching [33] or a traffic transfer technique via relay. Metadata is distributed via the EdgeHub–CloudHub tunnel. Thus, direct access to the cloud is not required, and by integrating the DNS server at the node layer, reliability can be maintained without access to the cloud CoreDNS when searching for services. EdgeMesh provides a load-balancing function using an Istio DestinationRule in the service. Typically, round-robin and random schemes are used. While the round-robin scheme distributes data equally, the random scheme randomly selects an endpoint and distributes data.

## 4. Local Scheduling Scheme in KubeEdge

This section discusses how the load-balancing algorithms such as round-robin and random schemes operate in KubeEdge. By defining the problem of KubeEdge’s load-balancing algorithms in an edge computing environment, we propose a local scheduling scheme to overcome the aforementioned problem and improve the throughput and latency in a KubeEdge-based edge computing environment.

### 4.1. KubeEdge’s Load-Balancing System

This subsection describes KubeEdge’s load-balancing system and its limitation. Generally, load balancing allows the distribution of the workload in an even manner among the available resources. Specifically, it aims to provide a continuous service in the event of a component failure by effectively provisioning application instances and resources. Furthermore, load balancing can reduce the task response time and optimize resource usage, thereby improving system performance at a reduced cost. Load balancing also offers scalability and flexibility for applications that may widen and require additional resources in the future.

**Figure 2:**
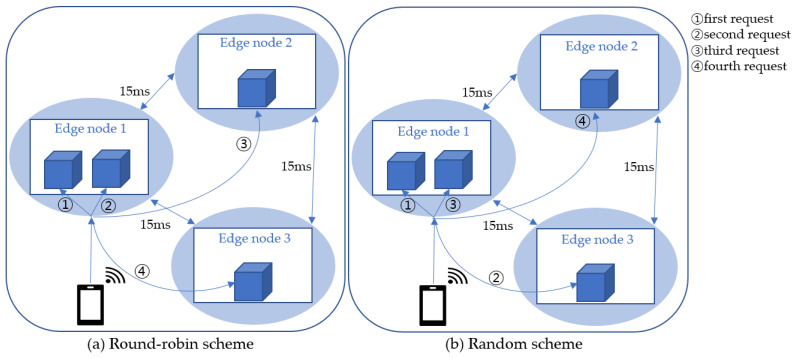
Load-balancing schemes in KubeEdge.

KubeEdge provides load balancing via EdgeMesh by distributing user requests equally across available pods. When the edge node receives user requests, it transmits them to EdgeMesh-Agent, which then distributes the traffic to the remote edge nodes according to the load-balancing policies. Round-robin in Figure 2a and Random in Figure 2b are the representative load-balancing algorithms used in EdgeMesh, and their functions are discussed as follows.

(a) Round-robin scheme: The round-robin scheme distributes user requests evenly among the pod resources. For example, in Figure 2a, four application pods are deployed to each Edge node 1, 2, and 3. Assuming that four user requests are received at Edge node 1, Edge node 1 will distribute the incoming requests evenly to each pod. Thus, the first and second requests are handled by the pods in Edge node 1, while the third and fourth requests are transmitted to pods of Edge nodes 2 and 3, respectively. 

(b) Random scheme: The random schedule distributes user requests randomly to any pod in the edge nodes. As shown in Figure 2b, the user requests received at Edge node 1 are distributed to individual pods throughout the cluster. For example, the first request is passed to the pod in Edge node 1, and the second request is passed to the pod in Edge node 3. Similarly, the third and fourth requests are passed to the pod in Edge nodes 1 and 2, respectively. It is interesting to note that the random scheme stochastically distributes traffic evenly to individual pods as the user traffic increases, which is similar to that in the round-robin scheme.

### 4.2. Problem Definition and Local Scheduling Scheme

In the load-balancing schemes in KubeEdge, the user traffic is evenly distributed regardless of the location of the edge node where the pod is placed. In other words, EdgeMesh in KubeEdge distributes the user traffic to the remote edge nodes without considering the delay in forwarding the requests. However, in an edge computing environment, the edge nodes are located far away from each other to cover a large-scale area, and the forwarding delay between the edge nodes is significant enough to degrade the throughput of the cluster. Therefore, we point out that load-balancing traffic to remote edge nodes degrades the performance of the KubeEdge cluster in an edge computing environment.

**Figure 3:**
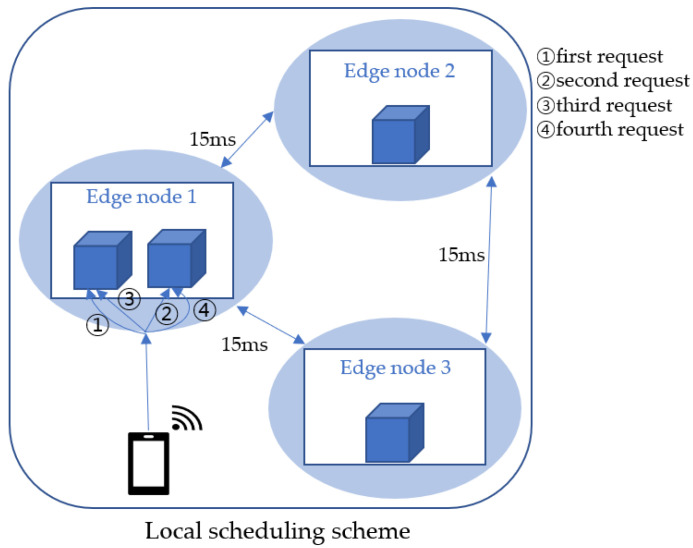
Local scheduling scheme in KubeEdge.

To solve the aforementioned problem, we propose a local scheduling scheme that processes user requests via pods located at the local node that receives them. In the local scheduling scheme, rather than transmitting the user requests to remote pods, they are distributed equally to the pods in the edge node that receive the user requests. For example, in Figure 3, four user requests are handled by two pods located at Edge node 1 without forwarding them to the pods in the remote edge nodes. In this way, the proposed scheme reduces the latency by preventing traffic forwarding between edge nodes in an edge computing environment and improves the throughput of the overall system by handling the user traffic immediately at the local edge nodes.

## 5. Performance Evaluations

In this section, we first describe the experimental setup of a KubeEdge-based edge computing environment. Then, we evaluate the performance of KubeEdge in terms of the number of pods of individual edge nodes, the pod distribution on edge nodes, and the delay between edge nodes by measuring the throughput and delay of individual edge nodes in increasing concurrent requests. We also compare the cumulative throughput and response time of the round-robin and local scheduling schemes to validate the feasibility of the local scheduling scheme in an edge environment.

### 5.1. Experimental Setups

**Figure 4:**
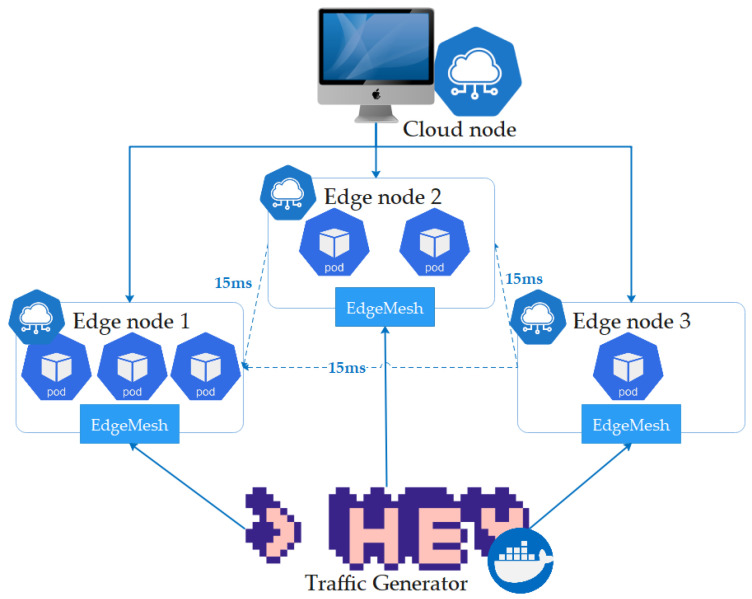
Experimental setup.

The KubeEdge clusters used for the performance evaluation consisted of one cloud node and three edge nodes, as shown in Figure 4. The cloud node runs with 4 central processing unit (CPU) cores and 8 GB of RAM, whereas edge nodes run with 4 CPU cores and 4 GB of RAM. Both nodes were installed with Docker version 20.10.14, KubeEdge version 1.9.1, Ubuntu 18.04.5, and Kubernetes API version 1.21.0 installed at a cloud node. The controllers provided a scheduler function by distributing the pods to the edge nodes; they were set to manually distribute the pods during the evaluation. 

The throughput was measured as the number of requests handled per second, and the response time was measured as the average time that individual requests are processed by the edge node, including the forwarding latency. The measurements were repeated 10 times to ensure that the results obtained were accurate, and an HTTP load-generator HEY tool [34] was used to generate the traffic.

### 5.2. Effect of Number of Pods

This subsection evaluates the effect of the number of pods with increasing concurrent requests. Notably, in this evaluation we focused on a single location (Edge node 1). While increasing the concurrent requests at Edge node 1 from 1 to 16, we measured the throughput and response time when the number of pods was 1, 2, and 4, respectively. 

**Figure 5:**
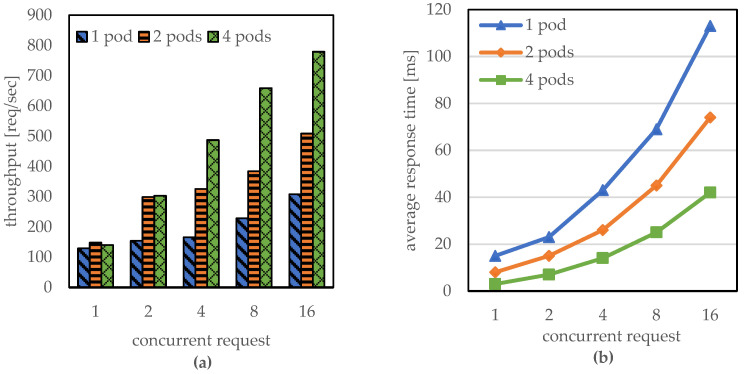
Performance at Edge node 1 with different numbers of pods: (**a**) throughput and (**b**) average response time.

As shown in Figure 5a, the throughput of Edge node 1 tends to increase as the incoming concurrent requests increase. However, it is noticeable that the throughput is bounded by a certain level with respect to the number of pods. For example, when the number of concurrent requests was 1, a throughput of approximately 139 req/s was noted, regardless of the number of pods; this observation indicates the ability of a single pod to handle the incoming user requests. When the number of concurrent requests was increased to 16, the maximum throughput of one pod was 308 req/s, whereas four pods could handle 779 req/s user requests. This indicates that an individual pod has its own capacity in terms of handling requests, and the throughput can be increased via cooperation with multiple pods. In addition, Figure 5b indicates that the average response time can be decreased by exploiting multiple pods in the edge node. For instance, the average response time decreased from 113 ms for one pod to 42 ms for four pods when the number of concurrent requests was 16.

### 5.3. Effect of Pod Distribution and Delay between Edge Nodes

We evaluated the effect of pod distribution on edge nodes as well as the delay between edge nodes while increasing the number of concurrent requests. To analyze the effect of pod distribution, we allocated different numbers of pods to three edge nodes. For example, 4-4-4 indicates that three edge nodes have the same number of pods, that is, 4 pods each, while 8-3-1 indicates that Edge nodes 1, 2, and 3 are allocated 8 pods, 3 pods, and 1 pod, respectively. For the evaluation, we increased the number of concurrent requests accessing Edge node 1 from 1 to 16. Notably, the incoming traffic at Edge node 1 is load-balanced to Edge nodes 2 and 3 by the EdgeMesh module at KubeEdge, where we used the round-robin scheme for load balancing in KubeEdge. It is noticeable that the random scheme has a similar tendency of traffic distribution with the round-robin scheme for high amounts of traffic from the stochastic point of view. Thus, both the round-robin and random schemes can distribute the incoming traffic to Edge nodes 1, 2, and 3 in a ratio of 4:4:4 when 4-4-4 pods are distributed on three nodes. Similarly, when pods are distributed in a proportion of 8-3-1, the incoming traffic is distributed to Edge nodes 1, 2, and 3 in a proportion of 8-3-1 because the round-robin scheme follows the policy of distributing the traffic evenly to each pod. To measure the effect of delay between edge nodes in an edge computing environment, we repeated the same evaluations by varying the delay between the edge nodes as 0, 15, and 30 ms. Since the traffic forwarded to the remote edge node is returned to Edge node 1 as a response, we measured the throughput and the average response time handled at Edge node 1 in a manner similar to that in the previous subsection.

**Figure 6:**
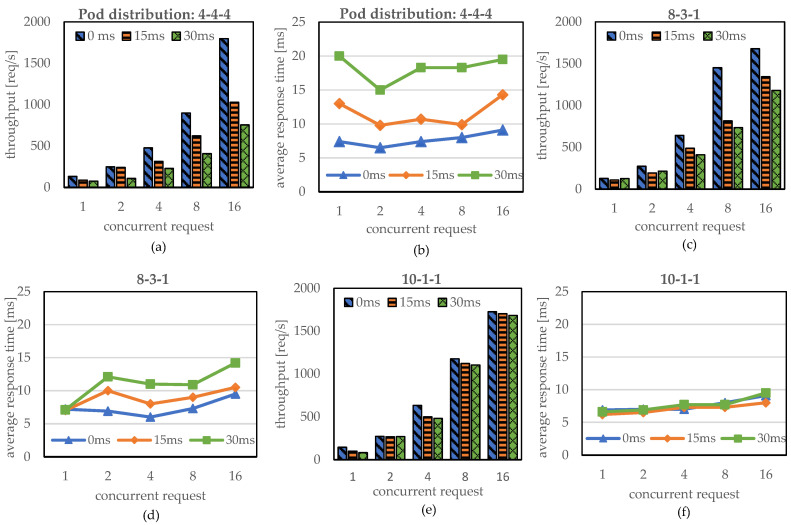
Throughput and average response time according to pod distribution and delay between edge nodes (**a**,**d**): 4-4-4, (**b**,**e**): 8-3-1, (**c**,**f**): 10-1-1.

Figure 6a–c present the throughput when the pod distribution to edge nodes is 4-4-4, 8-3-1, and 10-1-1, respectively, while Figure 6d–f show the corresponding average response times. In Figure 6a–c, there is no difference in throughput according to the pod distributions when the delay between edge nodes is 0 ms. For example, when the number of concurrent requests is 1, the throughputs are approximately 132 reqs/s, 127 reqs/s, and 145 reqs/s for the pod distributions 4-4-4, 8-3-1, and 10-1-1, respectively. When the concurrent request increases to 16, the throughputs increase to approximately 1796 reqs/s, 1677 reqs/s, and 1726 reqs/s, respectively. In addition, the response times in Figure 6d–f show steady response times of 6~9.5 ms according to the number of concurrent requests irrespective of pod distribution. Thus, we can conclude that the KubeEdge cluster can provide the same performance regardless of pod distribution in the case that there is no delay between edge nodes because there is no difference between handling traffic locally and remotely.

However, both the throughput and the average response time are highly affected by the pod distribution as the delay between edge nodes increases. For example, when the delay is 30 ms, the pod distributions 4-4-4, 8-3-1, and 10-1-1 show about 755 req/s, 1180 req/s, and 1682 req/s, respectively, for 16 concurrent requests. This indicates that the throughput degrades proportionally to the amount of traffic forwarded to the edge nodes. In other words, 2 out of 12 requests in the 10-1-1 pod distribution are forwarded to the remote edge nodes, whereas 8 out of 12 requests in the 4-4-4 pod distribution are handled by remote edge nodes. Therefore, more than 50% of the throughput was degraded in the 4-4-4 pod distribution, in contrast to that of the 10-1-1 pod distribution. Interestingly, this effect on the throughput degradation increases for higher delay between edge nodes and the response time. Figure 6d–f also show a similar tendency. The average response time in the 4-4-4 pod distribution is approximately 10~14.5 ms for a 15 ms delay, and it increases to 15~20 ms for a 30 ms delay, while that in the 10-1-1 pod distribution does not have any significant difference for the delay between edge nodes. In summary, the important lesson is that although the load balancing of EdgeMesh is designed to efficiently utilize the pod resource deployed in the edge nodes, the throughput and the average response time can be degraded by the delay between edge nodes where they are geographically distributed in an edge computing environment. 

### 5.4. Effect of Load-Balancing Schemes

We evaluated the effect of the load-balancing schemes by comparing the round-robin scheme in EdgeMesh and the proposed local scheduling scheme. To analyze the performance in an edge computing environment, we used a different traffic distribution for each pod distribution. In detail, we used 4:4:4, 8:3:1, and 10:1:1 traffic distributions for 6-6-6, 12-5-1, and 16-1-1 pod distributions, where x-y-z represents the number of pod distributions for each edge node in the KubeEdge cluster and x:y:z denotes the traffic distribution accessing each edge node. We used 18 pods in the cluster and differentiated only the pod distribution. In the same way, 12 concurrent requests were generated, and the traffic distribution was designed to follow the pod distribution ratio to ensure that each edge node utilizes the pod resources fully. In addition, we set the delay between edge nodes at 15 ms.

**Figure 7:**
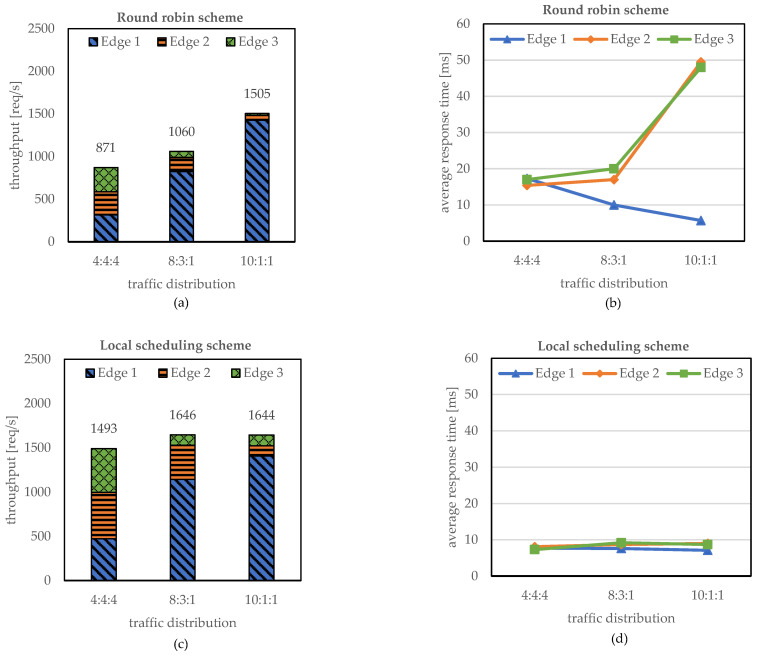
Throughput and average response time according to the load-balancing schemes (**a**,**c**): round-robin scheme, (**b**,**d**): local scheduling scheme.

Figure 7 presents the throughput and the response time of the round-robin and local scheduling schemes as the number of concurrent requests increases. As shown in Figure 7a, the throughput of the round-robin scheme shows 871 req/s for the 4:4:4 traffic distribution, while it achieves 1050 req/s for the 10:1:1 traffic distribution. This indicates that the throughput can be decreased by increasing the amount of traffic delivered to the remote edge nodes, as already discussed in the previous subsection. In the 4:4:4 traffic distribution, Edge node 1 forwards 12/18 of the incoming traffic to the remote edge nodes since the pods are distributed in the ratio of 6-6-6 to Edge nodes 1, 2, and 3. Similarly, Edge nodes 2 and 3 forward 12/18 of the incoming traffic to the remote edge nodes while handling the remainder of the traffic. In summary, Edge nodes 1, 2, and 3 forward 4×12/18, 4×12/18, and 4×12/18 incoming traffic to remote edge nodes in the 4:4:4 traffic distribution. However, the 10:1:1 traffic distribution is evaluated using a 16-1-1 pod distribution to each edge node, and Edge nodes 1, 2, and 3 transmit 10×2/18, 1×17/18, and 1×17/18 of the incoming traffic to the remote edge nodes. Therefore, we can conclude that the 10:1:1 traffic distribution distributes less traffic to the remote edge nodes compared with the 4:4:4 traffic distribution. This leads to less degradation of the throughput compared with the case of the 4:4:4 traffic. The average response time in Figure 7c shows the traffic analysis results. While all three edge nodes in the 4:4:4 traffic distribution show a response time of approximately 17 ms, Edge node 1 in the 10:1:1 shows the lowest average response time of 6 ms with the 10×16/18 incoming traffic handled locally. On the other hand, Edge nodes 2 and 3 in the 10:1:1 traffic distribution show a response time of approximately 50 ms, because the 1×17/18 incoming traffic is handled by the remote edge nodes.

In the local scheduling scheme, all the incoming traffic is processed at the edge nodes that receive the traffic; thus, performance degradation due to traffic forwarding to remote edge nodes does not occur. As a result, the local scheduling scheme achieves high throughput regardless of the traffic distribution. It can be observed from Figure 7b that the 4:4:4, 8:3:1, and 10:1:1 traffic distributions achieve throughputs of approximately 1493, 1646, and 1644 req/s, respectively. It is also observed that local scheduling eliminates the request forwarding latency between the edge nodes, which results in a low response time of approximately 8 ms regardless of the traffic pattern. Therefore, the local scheduling scheme achieves the maximum throughput by handling all the incoming traffic using the local edge nodes in an edge computing environment where the edge nodes are geographically distributed.

## 6. Conclusions

KubeEdge is a representative open source–based edge computing platform that extends the core functionalities of Kubernetes to the edge. We conducted diverse performance evaluations of KubeEdge in an edge computing environment in terms of the throughput and response time according to the pod distribution and the delay between edge nodes. On the basis of an experimental analysis, we found out that traffic forwarding from load balancing can degrade the throughput of the cluster in an edge computing environment due to the geographical distribution between the edge nodes. To overcome this problem, we propose a local scheduling scheme that handles traffic using local edge nodes. The evaluation results show that the local scheduling scheme outperforms the round-robin scheme in terms of the cumulative throughput and response time, regardless of the traffic patterns. We expect that the local scheduling scheme will be used to optimize the performance of edge computing environments. In the future, we will study the dynamic resource orchestration to adjust the containerized resources according to traffic demand.

## Data Availability

Not applicable.
